# Closing in on the causes of host shutoff

**DOI:** 10.7554/eLife.20755

**Published:** 2016-09-19

**Authors:** Ian Mohr

**Affiliations:** Department of Microbiology and the NYU Cancer Institute, NYU School of Medicine, New York, United StatesIan.Mohr@nyumc.org

**Keywords:** virus infection, host shutoff, translation, virology, mRNA decay, Human

## Abstract

Influenza A virus suppresses the translation of host mRNA by selectively remodeling and dominating the pool of mRNA in infected cells.

**Related research article** Bercovich-Kinori A, Tai J, Gelbart IA, Shitrit A, Ben-Moshe S, Drori Y, Itzkovitz S, Mandelboim M, Stern-Ginossar N. 2016. A systematic view on influenza induced host shutoff. *eLife* **5**:e18311. doi: 10.7554/eLife.18311

When a virus enters a cell it relies on the molecular machinery of its host to help it replicate. In particular, the virus relies on the ribosomes in the host cell to translate viral messenger RNA (mRNA) into polypeptides. Many viruses also impair the translation of cellular mRNA, via a process termed “host shutoff”, in order to prevent the production of anti-viral, host defense proteins. For example, poliovirus does this by inactivating a translation factor that is required to load ribosomes onto host mRNAs, all of which have a type of "cap" called an "m^7^G cap". Moreover, while the translation of these mRNAs is being suppressed, host ribosomes are involved in the translation of poliovirus mRNA, which does not have a cap: this is possible without the translation factor because poliovirus mRNA has an internal entry site for ribosomes (Jan et al., 2016). However, the mechanisms responsible for host shutoff in viruses that have mRNAs with m^7^G-caps, such as influenza A virus, have remained enigmatic.

Numerous mechanisms have been proposed to explain host shutoff by influenza A virus (IAV). One of these involves the viral mRNAs “stealing” capped fragments originally thought to be derived from cellular pre-mRNAs, followed by extension into mRNA (Koppstein et al., 2015). Other mechanisms put forward include the preferential translation of viral mRNA (Park and Katze, 1995), the degradation of cellular mRNA (Beloso et al., 1992), inhibiting the formation of cellular pre-mRNA (Nemeroff et al., 1998), the degradation of cellular RNA polymerase II (Rodriguez et al., 2007), and the retention of host mRNA in the cell nucleus (Fortes et al., 1994).

Now, in eLife, Noam Stern-Ginossar of the Weizmann Institute of Science and colleagues – including Adi Bercovich-Kinori and Julie Tai as joint first authors – report how they have combined RNA sequencing (which measures the abundance of different mRNAs) with ribosome profiling (which gives a measure of mRNA translation or protein synthesis (Ingolia, 2016)) to produce a genome-wide map that illustrates changes in the abundance and translation of both host and viral mRNA across the IAV replication cycle (Bercovich-Kinori et al., 2016). Unexpectedly they found that host and viral mRNAs were both translated with similar efficiencies, indicating that viral mRNAs were not preferentially translated relative to host mRNAs. Instead, IAV-induced host shutoff primarily originates from a reduced abundance of cellular mRNA and from the high levels of viral mRNA in both the nucleus and cytoplasm. Fluorescence-based measurements confirmed these findings and revealed that the reduced abundance of cellular mRNA has its origins in the nucleus. This likely involves an RNA endoribonuclease called PA-X, which is encoded in the genome of IAV, stimulating the decay of cellular mRNA (Khaperskyy et al., 2016).

Significantly, Bercovich-Kinori et al. – who are based at the Weizmann Institute, the Chaim Sheba Medical Center and Tel-Aviv University – found that the sensitivity of host mRNAs to IAV-induced host shutoff was not uniform, and that certain host mRNAs resisted IAV-induced decay. For example, shorter and more GC-rich host mRNAs were less impacted by IAV-induced decay. Furthermore, host mRNAs encoding proteins that maintain vital cellular processes (such as the proteins that maintain oxidative phosphorylation) were less perturbed by IAV because such processes support the replication of the virus. Thus, IAV infection effectively sculpts the total pool of mRNA in virus-infected cells, allowing select host mRNAs that are important for virus replication to persist and be translated.

Exploring further Bercovich-Kinori et al. noticed that a number of host mRNAs – those that are translated when eIF2α, a subunit of eukaryotic initiation factor 2, is phosphorylated – were enriched on ribosomes in IAV-infected cells. The phosphorylation of eIF2α is a response to physiological stress, including viral infection, that restricts overall protein synthesis (Jan et al., 2016), while stimulating the translation of select mRNAs that are required for stress responses. The researchers found evidence for increased levels of eIF2α phosphorylation at 4 hours post infection, and for much reduced levels at 8 hours post infection. Thus, while eIF2α phosphorylation is traditionally antiviral, the changes observed during IAV infection illustrate how a virus might benefit from this stress-response program. It is conceivable, therefore, that other stress-responsive host mRNAs that encode antiviral functions might restrict the replication of IAV, albeit incompletely. We do not know how viral mRNAs are translated when the levels of eIF2α phosphorylation transiently increase, but the overwhelming abundance of viral mRNAs may simply enhance the likelihood of capturing ribosomes carrying unphosphorylated eIF2α.

The study by Bercovich-Kinori et al. has broad implications in infection biology. Host shutoff enforced by viral domination of the mRNA pool has the advantage of preserving the cellular machinery needed for the translation of mRNAs with m^7^G caps. Instead of inactivating cellular translation factors, or relying on the preferential translation of viral mRNA, the mRNA pool can be remodeled via mRNA decay. This could allow the virus to better take advantage of critical host processes. Strikingly, the genomes of herpesviruses, poxviruses and coronaviruses, which all produce m^7^G-capped mRNAs, also encode proteins that can stimulate mRNA decay and, therefore remodel the mRNA pool inside cells (Abernathy and Glaunsinger, 2015; Jan et al., 2016). The extent to which the domination of the mRNA pool by viral mRNA drives host shutoff, or if similar host mRNAs resist virus-induced mRNA decay in cells infected with these different viruses, remains to be explored. Likewise, the latest work also illustrates how remodeling of the mRNA pool by mRNA decay, coupled with new transcription, might rapidly reprogram gene expression, and therefore potentially impact the stress responses of uninfected cells to a range of physiological stimuli.

**Figure 1.**
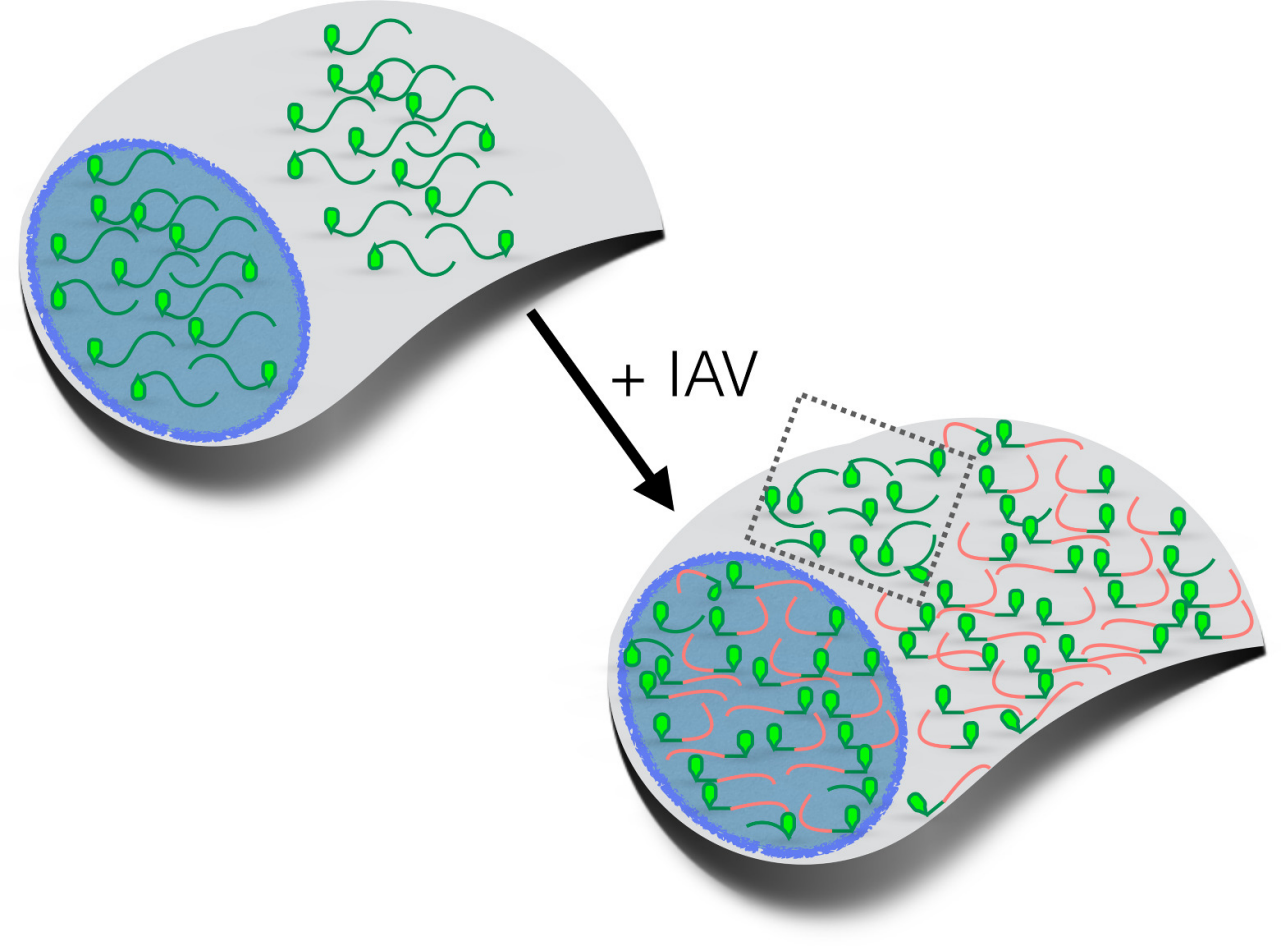
How influenza virus A drives virus-induced host shutoff. Top left: Pools of host mRNAs (green lines) in the nucleus (blue) and cytoplasm (grey) in uninfected cells. The 5' end of each mRNA has an m^7^G cap (light green filled). Lower right: Influenza virus A (IAV) mRNAs (*salmon lines*) dominate the mRNA pool in virus-infected cells. The 5’-end of viral mRNAs contain m^7^G-caps and 5’-proximal sequences derived from host RNAs. The abundance of host mRNAs within the nucleus is reduced by IAV: this is probably caused by an RNA endonuclease called PA-X. Some of the host mRNAs resistant to IAV-induced decay are shown within the cytoplasm (inside the dotted box): besides being shorter and more GC-rich than host mRNAs that are not resistant to IAV-induced decay, some of these mRNAs are stress-responsive and are translated when eIF2α is phosphorylated (see text). m^7^G: methyl-7-guanosine.
